# Protein painting reveals pervasive remodeling of conserved proteostasis machinery in response to pharmacological stimuli

**DOI:** 10.1038/s41540-022-00256-3

**Published:** 2022-11-28

**Authors:** Dezerae Cox, Angelique R. Ormsby, Gavin E. Reid, Danny M. Hatters

**Affiliations:** 1 Department of Biochemistry and Pharmacology, Bio21 Molecular Science and Biotechnology Institute, The University of Melbourne, Parkville, VIC 3010 Australia; 2 School of Chemistry, The University of Melbourne, Parkville, VIC 3010 Australia; 3 Present Address: Department of Chemistry, University of Cambridge, Cambridge, CB2 1EW United Kingdom

**Keywords:** Biochemical networks, Cell biology, Structural biology

## Abstract

The correct spatio-temporal organization of the proteome is essential for cellular homeostasis. However, a detailed mechanistic understanding of this organization and how it is altered in response to external stimuli in the intact cellular environment is as-yet unrealized. ‘Protein painting methods provide a means to address this gap in knowledge by monitoring the conformational status of proteins within cells at the proteome-wide scale. Here, we demonstrate the ability of a protein painting method employing tetraphenylethene maleimide (TPE-MI) to reveal proteome network remodeling in whole cells in response to a cohort of commonly used pharmacological stimuli of varying specificity. We report specific, albeit heterogeneous, responses to individual stimuli that coalesce on a conserved set of core cellular machineries. This work expands our understanding of proteome conformational remodeling in response to cellular stimuli, and provides a blueprint for assessing how these conformational changes may contribute to disorders characterized by proteostasis imbalance.

## Introduction

Precise spatio-temporal regulation of the proteome is essential for cellular homeostasis. This occurs at various levels, from the folding of individual protein domains, to binary protein–protein interactions, and the assembly of multi-protein macromolecular machines^1^. The result is the culmination of protein networks that drive biological functions^2^. Protein networks also link with other networks to mediate their regulation or to direct sequential functions (such as signaling pathways). A mechanistic understanding of cellular function and dysfunction in health and disease requires detailed knowledge of this organization and how it is altered in response to external stimuli^3^.

Quantitatively assessing the macromolecular organization of individual proteins in cells at the proteome-wide scale remains challenging. After decades of dedicated examination, the folding and stability characteristics of many individual proteins are well understood in vitro. High-throughput approaches to quantify protein conformation have included proteomic variations of these in vitro methodologies, relying on either the accessibility of protein regions to nonspecific proteases^4,5^, thermal aggregation-based methods^6,7^, or basal protein solubility^8,9^. A map of all possible binary protein–protein interactions is well developed in yeast^3^. However, these methods are all limited by the need to assess proteins either outside the cellular environment (i.e., ex vivo, post lysis) or under conditions of altered protein expression, and often cannot probe subtle changes in proteome organization within the undisturbed cellular context.

Protein painting methods have emerged as a way to gather conformational insight within intact cells at the proteome-wide scale^10–12^. One outstanding caveat of protein painting is the potential for chemical modification to induce distal conformational changes^13,14^, which necessitates conservative labeling regimes. Recently, we described the application of one such method based on a fluorogenic dye, tetraphenylethene maleimide (TPE-MI)^15^. TPE-MI reacts with exposed free cysteine thiols, which are the least surface-exposed residue of all amino acids in globular proteins, and provide an excellent target for examining protein conformation^16^. We have previously used TPE-MI to provide a snapshot of proteome conformation in live cells^15^, and proteome organization in response to denaturation in cell lysate^17^. In addition to widespread unfolding, TPE-MI can detect changes in the protein–protein interactome, including the binding of unfolded proteins by molecular chaperones. Here, we extend this methodology to explore remodeling of proteome networks in live cells in response to a cohort of commonly used pharmacological stimuli. We detect specific, heterogeneous responses to individual stimuli. We also find that the changes in proteome organization coalesce on a conserved set of core cellular machineries.

## Results

### Pharmacological stimuli induce changes in proteome conformation

**Fig. 1:**
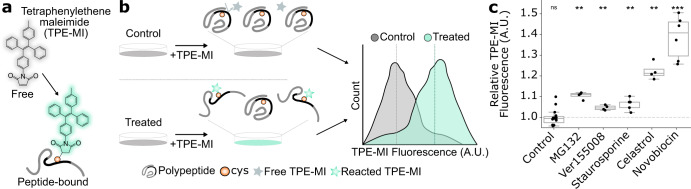
Pharmacological stimuli result in net increases in the TPE-MI reactivity of cellular proteins. **a** Structure of tetraphenylethene conjugated to a maleimide (TPE-MI). TPE-MI is inherently non-fluorescent in the free form, forming a fluorescent conjugate upon binding exposed thiol residues within polypeptides. **b** Method schematic for quantifying global proteome conformation. Neuro-2a cells were treated with MG132, VER155008, staurosporine, celastrol, novobiocin, or the vehicle control (milliQ water in the case of Novobiocin, else DMSO), before labeling with TPE-MI. Cells were then analyzed via flow cytometry. **c** Median TPE-MI fluorescence measured at 450 nm, normalized to the vehicle-treated control population. Shown are boxplots overlayed with individual datapoints of at least four biological replicates (dots; novobiocin *n* = 6, staurosporine *n* = 5, otherwise *n* = 4), *p < 0.05, **p < 0.01, ***p < 0.001 according to one-sample *t*-test against a hypothetical mean of 1. In **c**, individual points are overlayed on boxplots displayed as follows: center line corresponds to the median; box limits display upper and lower quartiles; and where shown, whiskers extend to the last or first data point that is within 1.5× the interquartile range of the box limits in the upper and lower directions, respectively. Source data are provided as a Source Data file.

To explore proteome remodeling in response to diverse pharmacological stimuli, we deployed TPE-MI in the mouse neuroblastoma cell line, Neuro-2a. As an immortalized neuron-like model, Neuro-2a cells are commonly used to investigate disrupted protein homeostasis in the context of neurodegenerative disease. TPE-MI is non-fluorescent when soluble, but becomes fluorescent upon conjugation with a thiol residue located in a molecular environment of sufficient rigidity to restrict the four phenyl rotamers of the TPE fluorophore (Fig. 1a). We selected a cohort of compounds to serve as pharmacological stimuli (distinct from physical stimuli such as heat shock or shear stress) that modulate different aspects of cellular homeostasis, and which act with varying degrees of specificity. The mechanism of action and relative specificity for these stimuli is summarized in Table 1.

**Table 1:** Pharmacological stimuli.

Compound	Target	Mechanism	Specificity	Source	Vehicle	Conc.	Incubation time (h)	Ref.
MG132	Proteasome	Binds the ß_5_ 20 S subunit	+++	Sigma #C2211-5MG	DMSO	10 µM	18 h	^53,54^
VER155088	HSP70	Binds the ATPase domain	+++	Sigma #SML0271	DMSO	20 µM	18 h	^8^^,55^
Staurosporine	Kinases	Non-selective ATP-competitive inhibitor	++	AbCam #ab146588	DMSO	500 nM	2 h	^56,57^
Celastrol	Heat shock response	Activation of HSF1 transcriptional regulation	+	Sigma #0869	DMSO	5 µM	18 h	^22,58,59^
Novobiocin	HSP90	Binds the ATP-binding site of the ATPase subunit	+	Sigma #N1628	mQ	800 µM	6 h	^26,55^

Compounds were diluted into fresh culture media before incubation at 37 °C. Compounds are categorized as poor (+), moderate (++), and high (+++) specificity according to the scope of the target and reported range of off-target effects.

Two stimuli were selected that have potent, reversible, and specific targets: the synthetic peptide aldehyde MG132 (Z-Leu-Leu-Leu-al;^18^), which inhibits the proteolytic activity of the proteasome by specific interaction with the ß_5_ (and at high concentration, the ß_1_) subunits of the 20 S proteasome; and the small molecule inhibitor VER155008, which inhibits the chaperone activity of Hsp70 family proteins by binding to the ATPase domain^19,20^. The third stimulus, staurosporine, was selected as a prototypical ATP-competitive kinase inhibitor that binds non-selectively to kinases with high affinity^21^. Thus, while still characterized by a specific mechanism of action, the target range of staurosporine is comparatively large. The final two stimuli were selected as having well-characterized broad-spectrum activities. Celastrol is often used as an inducer of the heat shock response due to its ability to activate HSF1^22^, however, it also has a range of off-target effects, including inhibition of the proteasome and HSP90 chaperones^23,24^. Novobiocin is an antibiotic for Gram-positive pathogens that inhibits bacterial DNA gyrase by binding the ATP-binding site in the ATPase subunit^25,26^. In mammalian cells, it has a lower level affinity to the C-terminal nucleotide-binding pocket of Hsp90, inhibiting its chaperone activity with an IC_50_ of ~700 µM^26–28^. Unlike other modifiers that target the N-terminal domain of Hsp90, novobiocin does not induce a heat shock response^29^. However, as a low-affinity Hsp90 inhibitor, it would be expected to have high levels of off-target activity^30,31^.

Cells stimulated with each compound, or the appropriate vehicle control (Table 1), were labeled in situ with TPE-MI for 30 min. Unreacted TPE-MI was then quenched with excess glutathione, which produces a non-fluorescent conjugate due to its inability to immobilize the phenyl rotamers of the TPE fluorophore^15^. Cells were harvested and immediately analyzed via flow cytometry (Fig. 1b). Cellular debris were excluded and the TPE-MI positive population was isolated (as described in ref. ^15^; the gating strategy is also summarized in Supplementary Fig. 1). The median fluorescence of the main cell population for treated cells was then normalized to the equivalent measurement in vehicle-treated control cells. Cells stimulated with each of the five compounds demonstrated significantly higher TPE-MI fluorescence relative to the control (Fig. 1c). This net increase in the global exposure of buried thiol residues suggests large-scale proteome rearrangements.

### Proteasome inhibition remodels protein complexes associated with apoptosis

**Fig. 2:**
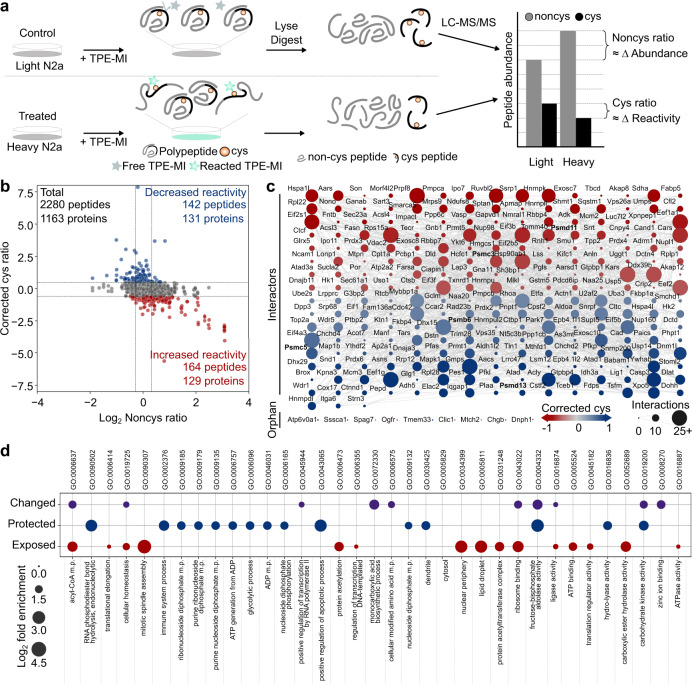
Conformational changes due to proteasome inhibition reflect cellular responses to MG132. **a** Method schematic for quantifying proteome conformation via proteomics. SILAC-labeled Neuro-2a cells were treated with pharmacological stimuli i.e., MG132 (heavy) or vehicle control DMSO (light), then labeled with TPE-MI and prepared for proteome analysis using LC-MS/MS. Peptide quantitation yielded the relative abundance of noncys-containing peptides (a measure of abundance) and cys-containing peptides (a measure of conformational status). **b** Representative scatterplot for processed noncys- and corrected cys-peptide abundance ratios in the presence of proteasome inhibitor MG132. Dots show per-peptide summary values across four biological replicates. Thresholds (red dotted lines) determined based on the control dataset are shown, outside which cysteine-containing peptides were considered more exposed (red) or more protected (blue). **c** Protein interaction network for changed peptides derived from **b**. Protein nodes are colored according to maximum corrected cys ratio and edges (lines) connect proteins with a known interaction (STRINGdb v 11.0, medium confidence score >0.4). Protein nodes are sized according to the number of interactions within the network. **d** Significantly enriched gene ontology terms (*p* < 0.05) for all proteins which changed reactivity (purple); or, more specifically, became protected (blue) or exposed (red) in **b**. Enrichment terms are filtered to minimize hierarchical redundancy (PantherGOSlim v 16.0). Source data are provided as a Source Data file.

We next assessed the contribution of individual proteins to global changes in proteome organization using proteomic analysis^15,17^ (Fig. 2a). We used stable isotope labeling by amino acids in cell culture (SILAC), whereby cells are differentially cultured in media containing unlabeled (light) or ^13^C l-Lysine and ^13^C,^15^N l-Arginine (heavy) to enable simultaneous comparison of proteins derived from treated and control cells. Briefly, SILAC-labeled Neuro-2a cells treated with either the vehicle control (light) or the stimulus of interest (heavy) were labeled with TPE-MI and then subjected to LC-MS/MS. Changes in the reactivity of cysteine thiols in individual proteins were quantified using the ratio of cysteine-containing peptides between stimulated and control cells, after correcting for any change in total per-protein abundance according to the non-cysteine-containing peptides (Corrected cys ratio; Fig. 2a). The resultant change in reactivity of cysteine-containing peptides constitutes a reporter of changes in protein conformation. Finally, *p* value weighted scaling and data-driven thresholds were applied such that changes in corrected cysteine ratios outside the control thresholds were considered biologically of interest^17^. A detailed description of the correction and normalization process is provided (see Methods: Peptide identification and quantitation).

The cysteine thiol reactivity profile under conditions of MG132-mediated proteasome inhibition is shown in Fig. 2b (results for the remaining stimuli are summarized in Supplementary Fig. 2). Of the 2880 cysteine-containing peptides quantified, representing 1163 proteins, 306 were seen to change reactivity (Fig. 2b). As a whole, these proteins formed a densely connected protein–protein interaction network (Fig. 2c; STRINGdb enrichment test, *p* < 0.0001), and were enriched for machinery associated with regulating biological quality (gene ontology (GO):0065008) and cellular homeostasis (GO:0019725) (Fig. 2c). Of these, 164 cysteine thiols increased in reactivity; the corresponding 129 proteins were enriched for machinery associated with biosynthesis and protein production (Fig. 2d), including ribosome binding (GO:0043022), regulation of transcription DNA-templated (GO:0006355) and translation (GO:0006412). In contrast, 142 cysteine thiols, representing 131 proteins, decreased in reactivity. This decreased reactivity is consistent with cys protection arising from the remodeling of protein complexes in functional response to the stimulus^17^. Proteins exhibiting protection in response to MG132-mediated proteasome inhibition included three proteasome subunits (PSMC5, PSMB6 and PSMD13), and were enriched with machinery associated with positive regulation of apoptotic processes (GO:0043065) and cell death (GO:0010942) (Fig. 2d). This is consistent with cellular phenotypes previously observed in response to proteasome inhibition with MG132, including reconfiguration of transcription and translation^32,33^ and induction of apoptosis^34^.

### Proteome organization is fine-tuned across core cellular activities in response to pharmacological stimuli

**Fig. 3:**
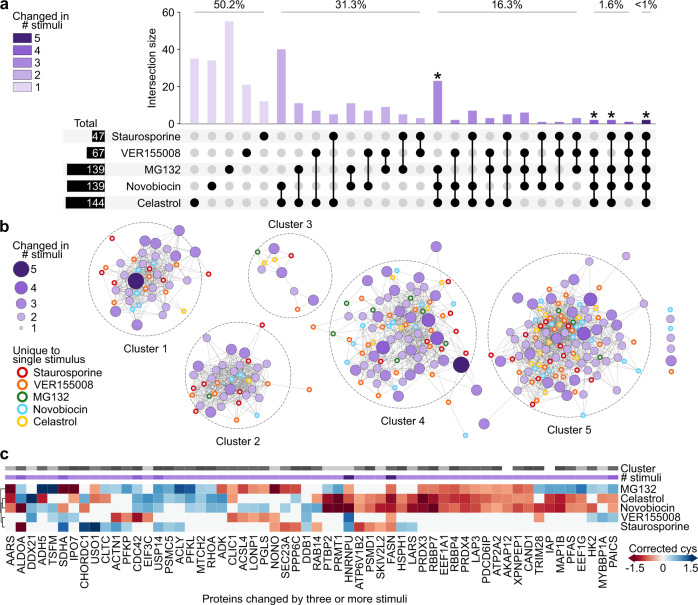
Conformational remodeling is poorly conserved in individual proteins in response to diverse stimuli. Proteins which were quantified in each of the five individual datasets, and which changed reactivity in response to at least one stimulus, were collected as the comparison protein set. **a** UpSet intersection plot of conformational change among comparison proteins. Individual proteins appear once within the column bar graph, according to the combination of stimuli in response to which they were seen to change conformation (irrespective of reactivity direction). The proportion of proteins associated with a change in 5, 4, 3, 2, or 1 conditions are indicated above the corresponding bars. **b** Protein interaction network for comparison proteins. Protein nodes are colored and sized according to their degree of commonality across stimuli in **a**, and for first-degree proteins, the corresponding stimulus is indicated by border color. Nodes were arranged organically following clustering with the Girvan–Newman community detection algorithm, and edges (lines) connect proteins with known interactions within each cluster (STRINGdb v 11.0, medium confidence score >0.4). **c** Heatmap for maximum change in cys reactivity among comparison proteins with degree >3 from **a**. Both degree (# stimuli) and cluster number are indicated for each protein above the heatmap, and the dendrogram shows the result of agglomerative clustering of the filtered comparison proteins in the stimulus dimension. Source data are provided as a Source Data file.

After stimulating cells with the remaining compounds (Table 1), a set of 646 proteins was quantified for all conditions and whose TPE-MI reactivities were altered by at least one stimulus (referred to herein as the comparison protein set). Comparison proteins were poorly conserved in their response to each stimulus (Fig. 3a). The reactivities of more than half of the comparison proteins were only altered in response to a single stimulus (Fig. 3a; first five bars totaling 50.2%). In contrast, less than 1% of the comparison proteins changed reactivity in response to all five stimuli (Fig. 3a; last bar), which is indicative of the highly distinct mechanisms by which the selected stimuli act on cells. The group containing celastrol, MG132 and novobiocin was unique; almost 10% of the comparison proteins were found to change reactivity in response to all three stimuli (Fig. 3a; * bars). This finding is consistent with the combined targeted and off-target effects of these stimuli intersecting. Together, this finding supports our ability to ascribe the response of individual proteins to distinct stimuli as opposed to merely measuring generic changes in a subset of proteins in response to any miscellaneous stimulus.

We next assessed the features of the comparison proteins. The protein–protein interaction network among these proteins was significantly more connected than would be expected by chance among a group of equivalent size (STRINGdb enrichment test, *p* < 0.0001). As with the proteasome inhibition experiment described above, this result suggests functional groupings within the comparison proteins. To further investigate this interaction network, we clustered the protein–protein interaction map using the Girvan–Newman fast greedy algorithm for community detection^35^. This produced five major clusters of densely connected proteins (Fig. 3b) and seven additional “orphan” proteins. Additional gene ontology analysis of proteins in each cluster revealed enrichment patterns reminiscent of core cellular activity hubs; namely, transcription (cluster 1), translation (cluster 2), intracellular trafficking (cluster 3), enzymatic activity and biosynthesis (cluster 4) and protein synthesis and degradation (cluster 5) (Supplementary Fig. 3). There was no discernable pattern of commonality in response to individual stimuli within the clusters (Fig. 3b). All five clusters contained proteins whose conservation ranged from two to at least four stimuli (purple nodes, where size indicates the number of stimuli in response to which a protein was found to have altered reactivity). In addition, those proteins whose conformational change was unique to a single stimulus were similarly spread across all five clusters (Fig. 3b; smallest nodes with outline colored according to the corresponding stimulus).

As well as the binary measure of conformational change, we also considered the maximum change in cysteine reactivity per-protein associated with individual stimuli (Fig. 3c and Supplementary Fig. 4). We observed an additional layer of heterogeneity in response to individual stimuli; i.e., proteins often became exposed in response to one stimulus but protected as a result of another. This finding was reminiscent of our previous study, in which heterogeneous changes in proteome solubility resulted from proteostasis imbalance^8^. However, despite identifying more than 90% identical proteins, there was no significant correlation between reactivity and solubility changes in any of the matched stimuli (MG132, novobiocin and VER155008; Supplementary Fig. 5). This result is consistent with the ability of TPE-MI to measure subtle changes in proteome organization when compared to an aggregation-based methodology^17^.

Together, these results demonstrate that TPE-MI is a sensitive measure of changes in conformation in response to pharmacological stimuli of varying specificity. For individual proteins, these changes were both subtle and poorly conserved across stimuli. However, at a proteome level, reorganization contributed to fine-tuning of five key hubs whose functions are known to be crucial for maintaining protein homeostasis.

### Conformational changes are consistent with remodeling of macromolecular complexes

**Fig. 4:**
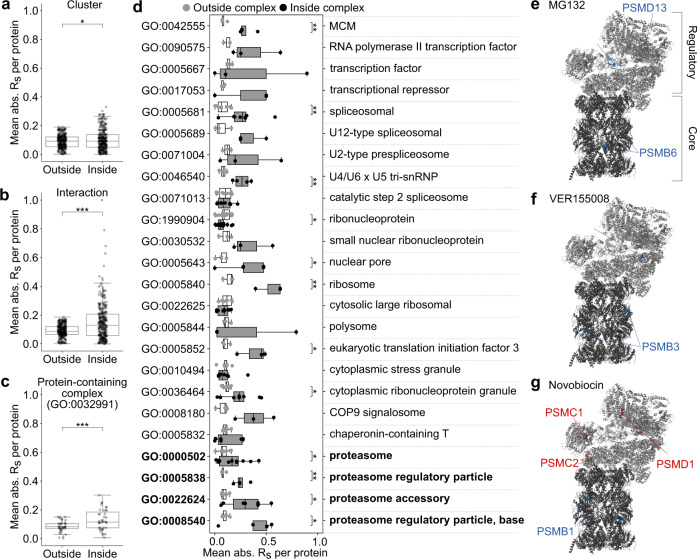
Unique conformational remodeling of macromolecular complexes is specifically associated with different stimuli. Correlation comparison for **a** functional clusters presented in Fig. 3, **b** interacting proteins, and **c**, **d** proteins annotated with gene ontology terms associated with macromolecular complexes. The pairwise correlation strength was calculated for all comparison proteins then binned according to whether one (outside) or both (inside) proteins identified with the feature of interest. The mean for each protein in both bins was then calculated and the resultant inside and outside values were compared according to a two-tailed *t*-test. *p < 0.05, **p < 0.01, ***p < 0.001. Terms related to the proteasome are highlighted in bold text. **e**–**g** Human 26 S proteasome ribbon structure adapted from PDB: 6MSB, consisting of the core 20 S proteasome (dark gray) and a single 19 S regulatory particle (light gray). Variations of the structure are presented for **e** MG132, **f** VER155008, and **g** novobiocin, whereby individual cysteine-containing peptides are colored according to their increase (red) or decrease (blue) in reactivity. The corresponding protein subunits are labeled with an equivalent color scheme. In **a**–**d**, individual points are overlayed on boxplots displayed as follows: center line corresponds to the median; box limits display upper and lower quartiles; and where shown, whiskers extend to the last or first data point that is within 1.5× the interquartile range of the box limits in the upper and lower directions respectively. Source data are provided as a Source Data file.

We further explored the per-protein heterogeneity by measuring the correlation between comparison proteins according to several grouping features. We found no correlation between the reactivity response of a protein and its degree of conservation across stimuli (spearman’s correlation coefficient = –0.092 for all datapoints; Supplementary Fig. 6). Similarly, there was no significant correlation among proteins associated with individual KEGG pathways (a collection of pathway maps containing molecular interactions, reactions, and relation networks responsible for cellular metabolism, structure, and information processing). However, the average magnitude of correlation (R_s_) for each protein with partners inside the same functional cluster was significantly different to those outside the cluster (Fig. 4a; two-tailed *t*-test, *p* = 0.032). Similarly, reactivity changes were significantly more correlated among protein interaction partners (Fig. 4b; two-tailed *t*-test, *p* < 0.001).

In addition to the presence of a one-to-one interaction between comparison proteins, we wondered if proteins associated with specific macromolecular complexes may be similarly correlated (Fig. 4c, d). We first considered proteins annotated with the generic gene ontology term “protein-containing complex” (GO: 0032991) and found that these proteins were significantly more correlated with each other than with non-complex proteins (Fig. 4c; two-tailed *t*-test, *p* < 0.001). Exploiting the hierarchical nature of gene ontology annotations, we then compared the average correlation among proteins for individual complexes which fall under the protein-containing complex umbrella (Fig. 4d). We identified several complexes for which reactivity changes were significantly correlated, including transcription, translation, and degradation machinery. This finding suggests at least some of the changes in reactivity we observe are a result of the assembly and/or disassembly of individual macromolecular complexes.

To explore whether subunits within a complex show correlated changes in cys reactivity, we examined the 26 S proteasome, which featured among the significantly correlated complex terms (proteasome (GO:0000502), regulatory (GO:0008540) and accessory (GO:0022624) particles; Fig. 4d bold). The structure of the human 26 S proteasome is well-characterized and conserved in eukaryotes, providing a scaffold onto which we could map observed changes in reactivity for individual cysteine thiol-containing peptides that resulted from different stimuli (Fig. 4e–g). The 26 S proteasome is comprised of two subcomplexes: the catalytic core complex (so-called 20 S proteasome) and one or two terminal activating regulatory particles (so-called 19 S particles)^36^. The core complex forms an enclosed cavity where catalytic threonine residues contributed by PSMB5, PSMB6, and PSMB7 (proteasome subunits beta type-5, -6, and -7, possessing chymotrypsin-like, caspase-like, and trypsin-like activity, respectively), degrade substrate. Subunits located within the core 20 S complex saw conserved protection in response to MG132, VER155008 and novobiocin (Fig. 4e–g). The observed protection is consistent with an increase in the occupation of the catalytic chamber, which we anticipate as a response to the accumulation of unfolded proteins.

The TPE-MI reactivity of the regulatory particle subunits was heterogeneous across stimuli. Member subunits of the 19 S regulatory particle (PSMC5, PSMD6 and PSMD13) were protected in the presence of MG132 and VER155008 (Fig. 4e, f). The regulatory particles associate with the termini of the barrel-shaped core complex, where they recognize ubiquitylated client proteins and assist in their unfolding and translocation into the *β*-ringed catalytic chamber. The increased protection in the presence of MG132 and VER155008 may reflect increased assembly and engagement of the 19 S regulatory particle with the surplus of core complexes present under basal conditions^37–39^ to enhance specific ubiquitin-mediated degradation of unfolded proteins. In addition, PSMC5 and PSMD6 are located at the interface between the lid and base regions of the regulatory particle, undergoing key conformational changes that facilitate switching between substrate-free and substrate-bound states of the proteasome^40^. In contrast, in the presence of novobiocin, three regulatory particle subunits (PSMC1, PSMC2, and PSMD1) became more reactive (Fig. 4g). This result is consistent with evidence that HSP90 is required for de novo assembly of the 26 S proteasome^41^, and that loss of HSP90 activity results in the disassembly of existing 26 S proteasomes^42^ followed by rapid dissociation of the regulatory particle components. Together, these examples demonstrate how seemingly heterogeneous changes in per-protein reactivity can reveal functional and specific remodeling of macromolecular complexes in response to different stimuli, even when the stimulus is expected to have a large extent of off-target activity.

## Discussion

Here, we demonstrate the application of TPE-MI to quantify conformational changes associated with a diverse range of pharmacological stimuli at the whole-cell and proteome-wide scales. Bulk measurements of increased TPE-MI reactivity associated with proteome unfolding were seen to mask subtle differences in reactivity at the per-protein level. Per-protein changes were largely unique to a specific stimulus. However, these changes occurred in a conserved set of functional machinery which broadly matches core cellular activities. These conserved hubs exhibited heterogeneous changes in response to different stimuli. Together, these results hint at finely tuned control of proteome conformation in response to perturbation that is commensurate with their degree of specificity.

We identified significant correlations among proteins known to interact. This enabled us to ascribe many of the observed changes in reactivity to the remodeling of protein–protein interactions, including within multi-subunit macromolecular complexes. The detailed structure-function information available for the human proteasome allowed us to rationalize the heterogenous changes observed for individual subunits of the 20 S core and 19 S regulatory particles. The ability to obtain mechanistic details for other macromolecular machines remains challenging. However, in addition to existing structural models the advent of machine-learning approaches such as AlphaFold-multimer^43^, which enable the prediction of protein–protein interaction interfaces from protein sequence alone, holds promise. These advances will assist in evaluating our observations of potential binding/unbinding events in silico to direct in vitro and in vivo validation efforts.

As Luck and colleagues observe^3^, it remains infeasible to assemble a map of proteome organization in the many thousands of physiological and pathological cellular contexts by systematically identifying endogenous protein–protein interactions (PPIs). However, the data reported here demonstrate the potential for protein painting technologies such as TPE-MI to provide detailed inventories of remodeling events that occur in response to stimuli within the intact cellular environment. TPE-MI fluorescence in response to these (or similar) stimuli has also been characterized in a number of other immortalized and primary cell lines, including HeLa, primitive neural stem cells, bone marrow and hematopoietic stem cells^15,44^. Moreover, several TPE-MI derivatives have now been developed, expanding the toolbox available for protein painting applications^45,46^.

There are two limitations of this method; namely, the inability to monitor proteins which don’t contain a free cysteine thiol residue, and the failure to adequately quantify some proteins across all stimuli, which meant they were subsequently removed from the comparison dataset. Both of these will be readily addressed by combining TPE-MI with complementary protein painting strategies, for example, lysine modification^12^, and by leveraging ongoing advancements in the data-independent acquisition and quantitation methodologies^47^. This work expands our understanding of proteome conformational remodeling in response to cellular stimuli and provides a blueprint with which to assess how these conformational changes may contribute to disorders characterized by proteostasis imbalance.

## Methods

### Materials

All materials used in this study were purchased from Sigma-Aldrich (St. Louis, MO, USA) unless otherwise indicated. The mouse neuroblastoma cell line Neuro-2a (N2a) was obtained from lab cultures originating from the American Type Culture Collection, and cultures were routinely screened for mycoplasma contamination. TPE-MI was a kind gift from Dr Yuning Hong (La Trobe University), and stocks prepared at 10 mM in DMSO were stored in the dark at 4 °C before use. All work was completed with protein low-bind plastics unless otherwise indicated.

### Cell culture

Neuro-2a cells were cultured in Dulbecco’s modified Eagle’s medium (DMEM; Thermo Fischer Scientific) supplemented with 10% (v/v) fetal bovine serum (Thermo Fischer Scientific) and 1 mM l-glutamine (Thermo Fischer Scientific). In the case of isotopically labeled cultures (SILAC), cells were cultured in DMEM (Silantes) supplemented with either unlabeled (light) or ^13^C l-Lysine and ^13^C,^15^N l-Arginine, along with 10% (v/v) dialyzed fetal bovine serum (Thermo Fischer Scientific) and 1 mM l-glutamine (Silantes). To ensure complete incorporation of labeled amino acids, cells were cultured for at least 8 doublings prior to use. Cells were maintained at 37 °C in a humidified incubator with 5% CO_2_ and were reseeded into fresh culture flasks once at 80% confluency following dissociation with 0.05% (w/v) trypsin-EDTA in PBS. For plating, cell count and viability were automatically determined using a Countess trypan blue assay (Thermo Fischer Scientific).

### Pharmacological stimuli and TPE-MI labeling

Cells were seeded at 40% confluency into either 25 cm^2^ culture flasks or six-well plates and cultured overnight. In the case of SILAC-labeled cells, compounds were prepared in fresh heavy-labeled media, and the appropriate vehicle control in an equivalent volume of unlabeled media. Culture media was removed and replaced with treatment media, after which cells were incubated at 37 °C in a humidified incubator with 5% atmospheric CO_2_. Details of the concentration and duration for each compound are presented in Table 1.

Following incubation, media was removed and replaced with a half-volume of fresh serum-free media (either unlabeled or labeled as appropriate) containing TPE-MI to a final concentration of 100 µM. Cells were incubated for 30 min, then immediately washed with 3× excess of PBS containing 10 mM Glutathione to react any remaining TPE-MI. Cells were then washed with PBS, mechanically detached using a cell scraper and centrifuged at 300 × *g* for 5 min.

### Flow cytometry

For flow cytometry, cells were resuspended in PBS and analyzed using an LSRFortessa flow cytometer (BD Biosciences)^15^. Briefly, between 10,000−60,000 events were collected at a high flow rate using a forward scatter threshold of 5000. Pulse area, height and width data were collected with the 355 nm laser and a 450 ± 50 nm bandpass emission filter. The data were analyzed with FlowJo (Tree Star Inc.) using the gating strategy summarized in Supplementary Fig. 1. The median TPE-MI fluorescence was then exported for TPE-MI + cells. The average TPE-MI fluorescence across vehicle control replicates was used to normalize the corresponding compound-treated sample measurements, and the resultant normalized data were subjected to a one-sample t-test against a hypothetical mean of 1.

### Sample preparation for mass spectrometry

Cell pellets for proteomics were lysed by resuspension in lysis buffer (150 mM NaCl, 50 mM Tris, pH 8.0, 1% (v/v) IGEPAL CA-630, 0.5% (w/v) sodium deoxycholate, 0.1% (w/v) sodium dodecyl sulfate) containing cOmplete Mini, EDTA-free Protease Inhibitor Cocktail and 250 U benzonase and incubated on ice for 30 min. The lysate was spun at 20,000 × *g* for 30 min to pellet cellular debris and the supernatant was collected in a fresh tube. Protein concentration was determined via bicinchoninic acid protein assay (BCA; Thermo Fischer Scientific) using bovine serum albumin as the mass standard. In the case of isotopically labeled cultures, protein from each control and treated sample was combined 1:1 (w/w). Prepared lysates were then precipitated via dilution into a fivefold excess of ice-cold 100% acetone and incubated at –20 °C overnight.

Samples were centrifuged at 20,000×*g* for 30 min at 4 °C, then the supernatant was discarded. Protein pellets were solubilized in 100 µl of 8 M urea in 50 mM triethylammonium bicarbonate (TEAB), and incubated with shaking at 37 °C for 45 min. Proteins were reduced using 10 mM tris(2-carboxyethyl)phosphine, pH 8.0, and alkylated with 10 mM iodoacetamide for 45 min, before being digested with 2 µg trypsin (Thermo Fischer Scientific) overnight with shaking at 37 °C. Peptides were then desalted via solid-phase extraction using an Oasis HLB 1 cc Vac Cartridge (catalog number 186000383, Waters Corp., USA) washed with 1 ml of 80% (v/v) acetonitrile (ACN) containing 0.1% v/v trifluoroacetic acid (TFA), then pre-equilibrated with 2.4 ml of 0.1% (v/v) TFA. Peptides were acidified with formic acid to a final concentration of 1% (v/v), then loaded onto the cartridge and washed with 1.5 ml of 0.1% (v/v) TFA before being eluted in 800 µl of 80% (v/v) ACN containing 0.1% (v/v) TFA. Samples were collected in fresh tubes and lyophilized (VirTis Freeze Dryer, SP Scientific). Peptides were resuspended in 80 µl distilled water and quantified using a BCA assay as above. Peptide aliquots were combined with 5× loading buffer to yield 20 µl containing 2 µg peptides in 2% (v/v) ACN containing 0.05% (v/v) TFA for analysis.

### NanoESI-LC-MS/MS

Samples were analyzed by nanoESI-LC-MS/MS using an Orbitrap Fusion Lumos mass spectrometer (Thermo Scientific) fitted with a nanoflow reversed-phase-HPLC (Ultimate 3000 RSLC, Dionex). The nano-LC system was equipped with an Acclaim Pepmap nano-trap column (Dionex—C18, 100 Å, 75 µm × 2 cm) and an Acclaim Pepmap RSLC analytical column (Dionex—C18, 100 Å, 75 µm × 50 cm). For each LC-MS/MS experiment, 0.6 µg of the peptide mix was loaded onto the enrichment (trap) column at an isocratic flow of 5 µl min^−1^ of 3% CH3CN containing 0.1% (v/v) formic acid for 5 min before the enrichment column was switched in-line with the analytical column. The eluents used for the LC were 0.1% (v/v) formic acid (solvent A) and 100% ACN/0.1% formic acid (v/v) (solvent B). The gradient used (300 nl min^−1^) was from 3–22% B in 90 min, 22–40% B in 10 min and 40–80% B in 5 min, then maintained for 5 min before re-equilibration for 8 min at 3% B prior to the next analysis. All spectra were acquired in positive ionization mode with full scan MS from m/z 400–1500 in the FT mode at 120,000 mass resolving power (at m/z 200) after accumulating to a target value of 5.00e^5^ with a maximum accumulation time of 50 ms. Lockmass of 445.12002 was used. Data-dependent HCD MS/MS of charge states >1 was performed using a 3 s scan method, at a target value of 1.00e^4^, a maximum accumulation time of 60 ms, a normalized collision energy of 35%, an activation Q of 0.25, and at 15,000 mass resolving power. Dynamic exclusion was used for 45 s.

### Peptide identification and quantitation

Initial identification was carried out using MaxQuant (version 1.6.2.10) against the Swissprot Mus Musculus database (Version: 2016_06; 16794 entries). The search was conducted with 20 ppm MS tolerance, 0.6 Da MS/MS tolerance, with one missed cleavage allowed and a match between runs enabled. Variable modifications included methionine oxidation, N-terminal protein acetylation, and N-terminal methionine cleavage, while carbamidomethylcysteine was set as a fixed modification. The false discovery rate maximum was set to 0.005% at the peptide identification level (the actual was 0.005 for each replicate) and 1% at the protein identification level. All other parameters were left as default.

The change in cysteine peptide abundance following TPE-MI labeling was then determined via custom python scripts (available from DOI: [10.5281/zenodo.6548917]). The logic was as follows; first, the common contaminant protein keratin was removed. Then, quantified proteins were filtered to those identified by at least two unique peptides, at least one of which contained a cysteine residue. The average peptide abundance for the non-cysteine-containing peptides was then calculated for each protein. These values were used to normalize the cysteine-containing peptides, yielding a corrected cys ratio which accounts for any changes in overall protein abundance due to a given stimulus.

The resultant corrected cysteine and non-cysteine ratios were then scaled using a *p* value weighted correction, as described previously^17^. In essence, rather than using the *p* value as an arbitrary cut-off, this method scales the mean of biological replicates (*n* = 3) according to the relative confidence with which it deviates from the expected value (in this case 0). We then applied a set of thresholds for the cysteine and non-cysteine peptides derived from a control experiment in which both the light- and heavy-labeled were treated with the vehicle control DMSO (Supplementary Fig. 7). The thresholds were calculated to contain 95% of the control dataset (corresponding to a *z*-score of 1.96), and datapoints outside these thresholds are considered a response to the stimulus. To compare different stimuli, only proteins quantified according to the above criteria (pre-thresholding) in all conditions were considered. From the resultant list of proteins, the comparison set contained those for which at least one cysteine-containing peptide exceeded the control threshold in at least one condition. Finally, a summary measure was calculated as the maximum corrected cys ratio per-protein in response to each stimulus, which was then used for subsequent protein-based comparisons.

### Functional characterization

Physicochemical properties for individual cysteine peptides and proteins of interest were compiled from various databases, including the Protein Data Bank (https://www.ebi.ac.uk/pdbe/), and STRINGdb (v 11.0, medium confidence score >0.4;^48^) via Cytoscape v3.9.0^49^. Gene ontology annotations for individual proteins were collected from UniProt (https://www.uniprot.org/). Gene ontology enrichment analyses were completed using PantherGOSlim (http://pantherdb.org;^50^) against the background of all proteins identified in the raw dataset. Significantly enriched terms were filtered according to *p* < 0.05, and the most specific terms from each hierarchically redundant family are presented. Connected clusters were detected in the protein–protein interaction map using the Girvan–Newman fast greedy algorithm^35^ for community detection, as implemented by the cytoscape Glay plugin^51^. To compare potential sources of correlation among individual proteins, a series of feature bins were considered; namely, community cluster, KEGG pathways, protein–protein interactions, and complex memberships. The correlation strength between individual proteins was determined as the absolute Spearman’s correlation coefficient (R_s_) for individual protein pairs, and each pair was then binned according to whether one (outside) or both (inside) proteins identified with the feature of interest. Pairs for which neither protein identified with the feature of interest were discarded. For each protein, the mean correlation inside and outside the feature bin was then calculated. Finally, for features associated with at least 3 proteins, the mean correlation across all feature proteins was compared.

### Comparison to Sui et al. dataset

Summary data from Sui et al.^8^ was downloaded from the supplementary information available online [10.1073/pnas.1912897117]. Datasets for stimuli common to both studies were collected (MG132, VER155008 and novobiocin), and the pellet-based solubility ratio (Sui dataset) was compared with the maximum corrected cysteine ratio (TPE-MI dataset) for individual proteins. Proteins altered in response to the stimuli in both datasets were collected, and in cases where more than three proteins passed this filter, their correlation was assessed via linear regression.

### Reporting summary

Further information on research design is available in the Nature Research Reporting Summary linked to this article.

## Supplementary information

Supplementary Material

Reporting Summary

## Data Availability

The source data underlying Figs. 1c, 2b-d, 3a, 3c, and 4a-d are provided as a Source Data file. The mass spectrometry proteomics data have been deposited to the ProteomeXchange Consortium via the PRIDE63 partner repository with the dataset identifier PXD033152. Protein structures presented in Fig. 4 are available via the PDB: 6MSB [10.2210/pdb6msb/pdb]. Preprocessed datasets for proteomics and flow cytometry are also available from zenodo via [10.5281/zenodo.6439170].

## Source data

Source Data
Supplementary Data 1

## Code availability

Statistical analyses were performed using either the scipy module in python^52^ or GraphPad Prism (v 8.4.3). The exact *p* values and statistical details are provided in Supplementary Data 1. All analysis scripts are available from zenodo via the [10.5281/zenodo.6548917].
