# Supplementary material for: Protein painting reveals pervasive remodeling of conserved proteostasis machinery in response to pharmacological stimuli
Source: NPJ Syst Biol Appl. 2022 Nov 28;8:46. doi: 10.1038/s41540-022-00256-3 (PMC9705707; doi:10.1038/s41540-022-00256-3)
Supplement: Supplementary file 1 — Supplementary Material [file 41540_2022_256_MOESM1_ESM.pdf]

Supplementary Information for:

Protein painting reveals pervasive remodeling of conserved proteostasis machinery in response to pharmacological stimuli.

Dezerae Cox[1, 2, *], Angelique R. Ormsby[1], Gavin E. Reid[1,3], Danny M. Hatters[1, *]

[1] Department of Biochemistry and Pharmacology, Bio21 Molecular Science and Biotechnology Institute, The University of Melbourne, Parkville, VIC 3010, Australia

[2] Current address: Department of Chemistry, University of Cambridge, Cambridge CB2 1EW, United Kingdom

[3] School of Chemistry, The University of Melbourne, Parkville, VIC 3010, Australia

* Corresponding authors: D. Cox (dc597@cam.ac.uk), D.M. Hatters (dhatters@unimelb.edu.au)

This PDF file includes:

Supplementary Figure 1: Flow cytometry gating strategy. Relates to Fig. 1.

Supplementary Figure 2: Summary of cysteine thiol reactivity changes associated with individual pharmacological stimuli. Relates to Fig. 2.

Supplementary Figure 3: Gene ontology enrichment for each protein-protein interaction cluster. Relates to Fig. 3.

Supplementary Figure 4: Per-protein changes in cysteine reactivity are heterogeneous. Relates to Fig. 3.

Supplementary Figure 5: Correlation between cysteine thiol reactivity and solubility changes measured in MG132, novobiocin or VER155008.

Supplementary Figure 6: Correlation between conservation degree and maximum cysteine thiol reactivity change per protein. Relates to Fig. 4.

Supplementary Figure 7: Threshold derivation from control dataset.

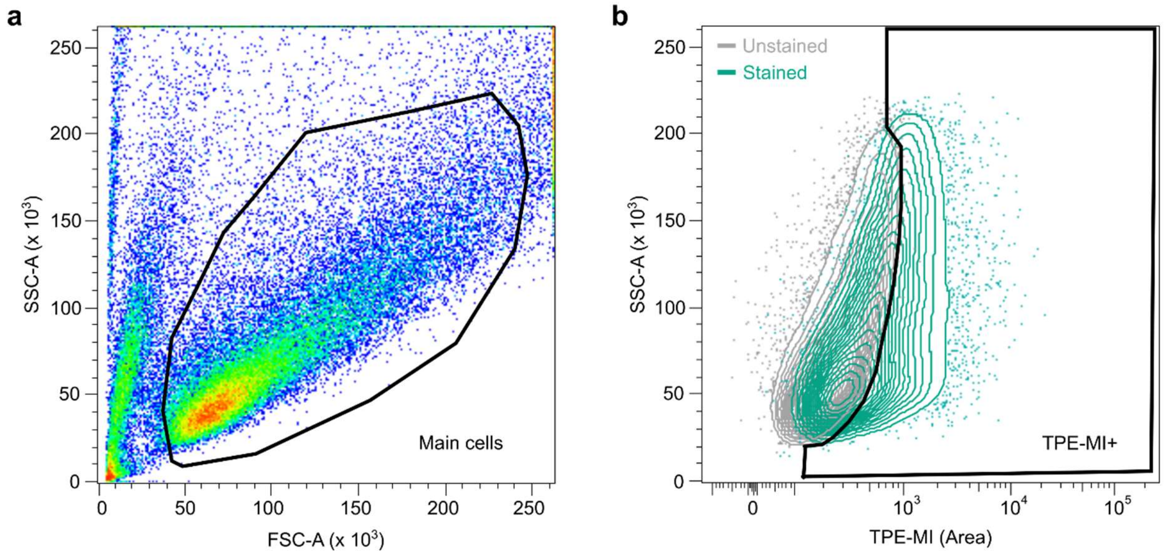

Supplementary Figure 1: Flow cytometry gating strategy. Representative gates are shown for (***a***) forward and side scatter selection of the main cells population and (***b***) cells containing higher than background TPE-MI fluorescence.

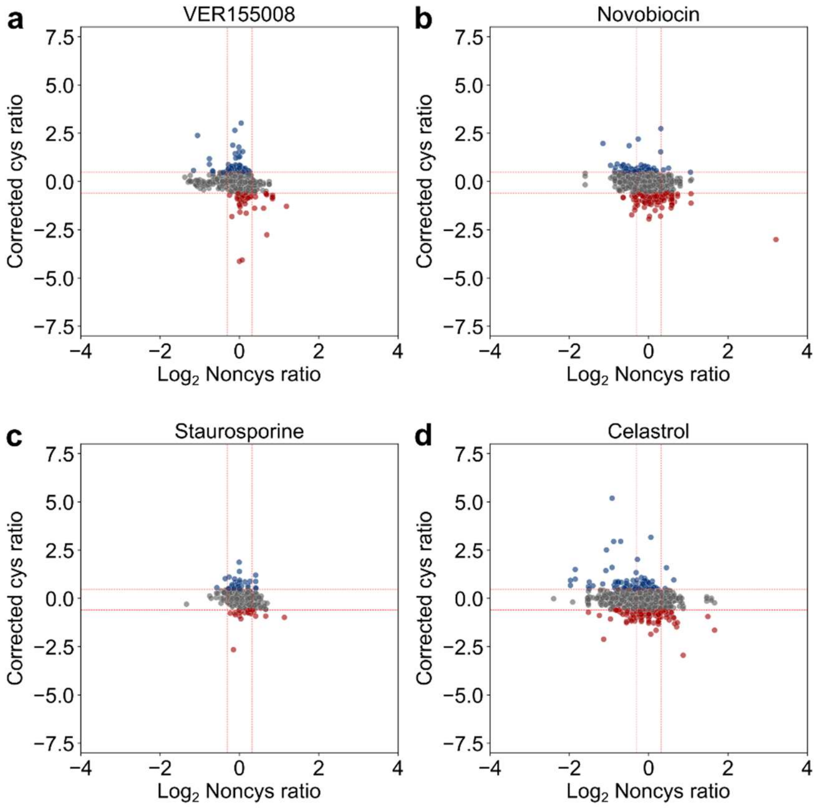

<u>Supplementary Figure 2:</u> Summary of cysteine thiol reactivity changes associated with individual pharmacological stimuli. Scatterplots for processed noncysteine- and cysteine-peptide ratios in the presence of (***a***) VER155008, (***b***) novobiocin, (***c***) staurosporine, and (***d***) celastrol. Dots represent per-peptide summary value across four biological replicates. Thresholds (red dotted line) determined based on control dataset, outside which cysteine-containing peptides are considered to be more exposed (red) or more protected (blue) in response to the specific stimulus.

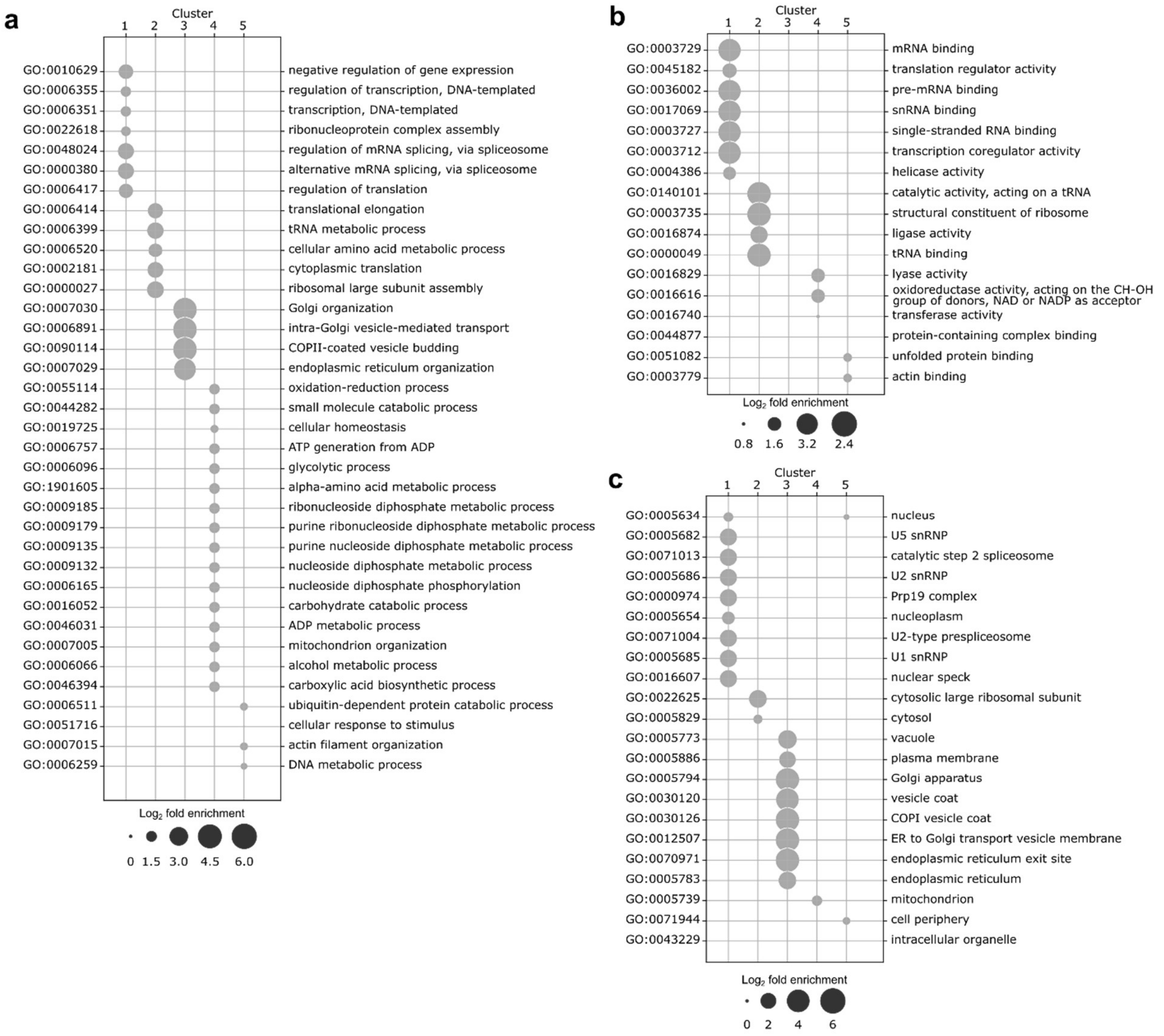

<u>**Supplementary Figure 3:**</u> **Gene ontology enrichment for each protein-protein interaction cluster**. Significantly enriched gene ontology terms ($p < 0.05$) for proteins found in functional clusters 1 - 5. Independent search results are shown for (***a***) biological process, (***b***) molecular function or (***c***) cellular component, and enrichment terms were filtered to minimize hierarchical redundancy among ontology families (PantherGOSlim v 16.0).

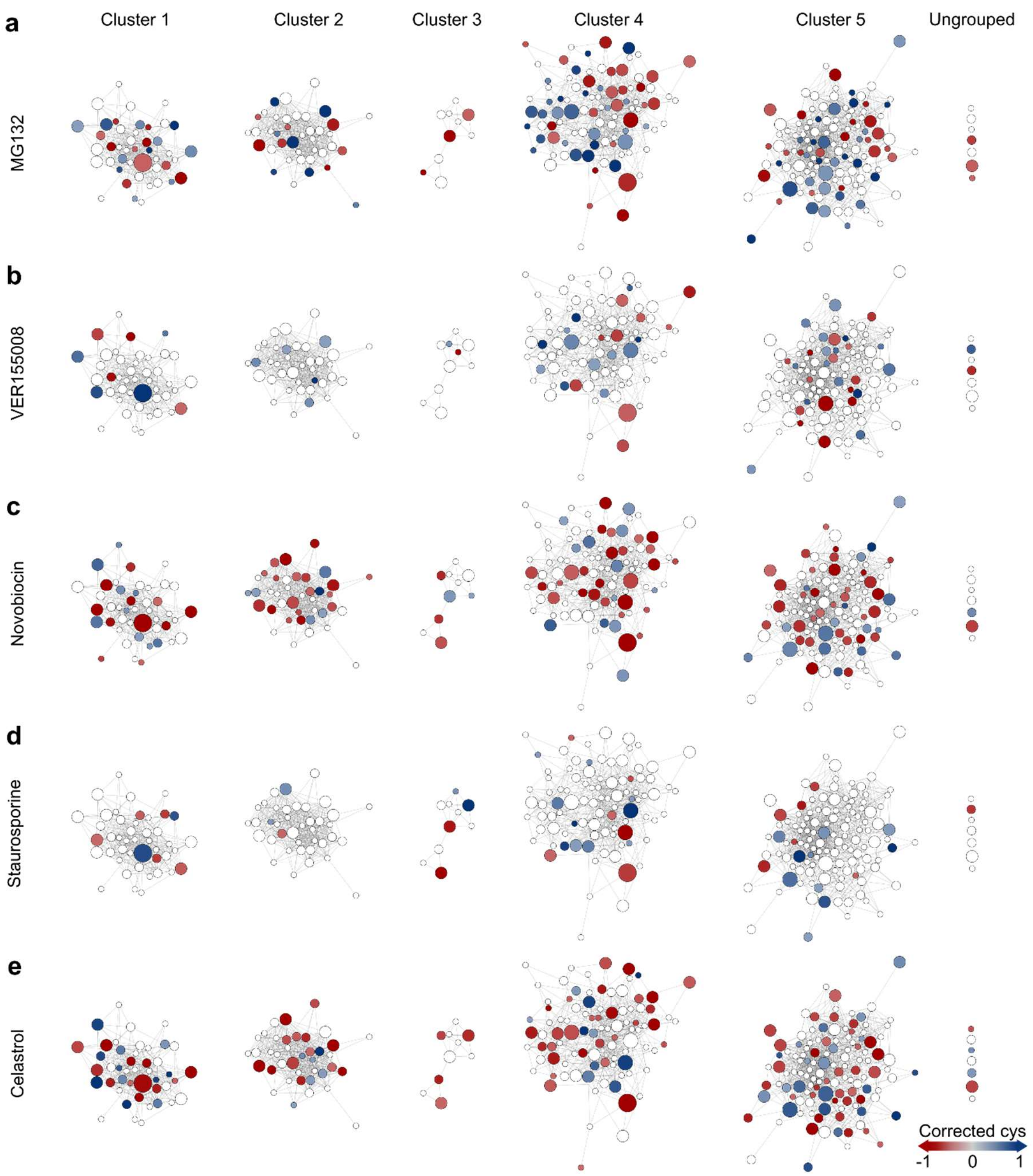

Supplementary Figure 4: Per-protein changes in cysteine reactivity are heterogeneous. Clustered protein interaction network for comparison proteins. Protein nodes are sized according to degree of commonality across stimuli and colored according to maximum corrected cysteine thiol ratio change in response to each individual stimulus, (***a***) MG132, (***b***) VER155008, (***c***) novobiocin, (***d***) staurosporine and (***e***) celastrol. Nodes were arranged organically following clustering with Girvan-Newman community detection algorithm, and edges (lines) connect proteins with known interactions within each cluster (STRINGdb v 11.0, medium confidence score > 0.4).

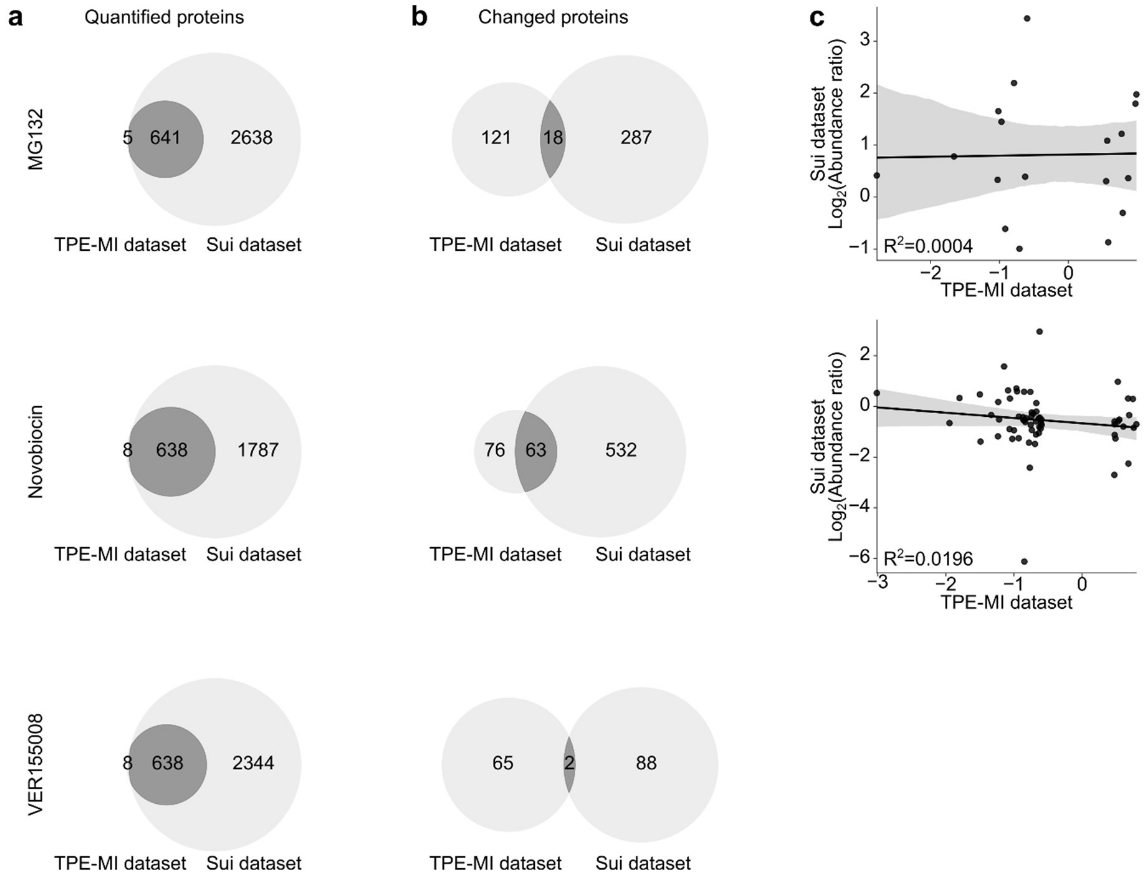

<u>Supplementary Figure 5:</u> **Correlation between cysteine thiol reactivity and solubility changes measured in MG132, novobiocin or VER155008**. The overlap between proteins (***a***) identified or (***b***) significantly changed according to the Sui *et al.* dataset [8] compared with the comparison set of proteins (TPE-MI dataset) quantified in the present study. (***c***) Correlation between the Sui *et al.* and TPE-MI datasets in which more than three common proteins were identified as having undergone a significant change due to stimulation with either MG132 or VER155008. Confidence interval derived via automatic bootstrap estimation of the linear regression.

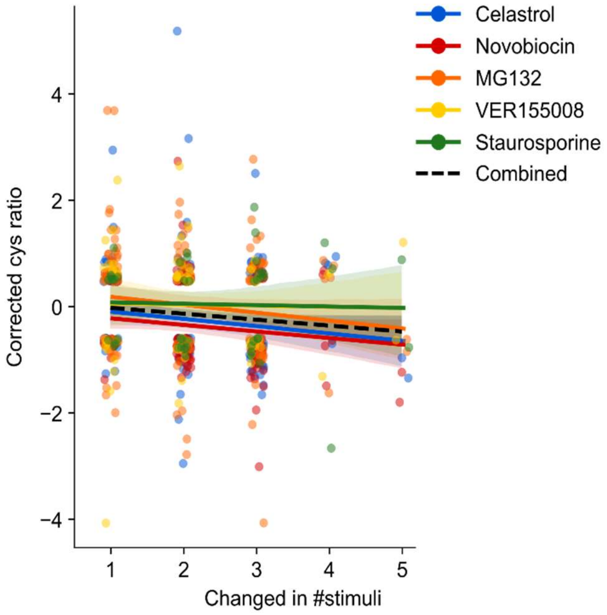

<u>Supplementary Figure 6:</u> **Correlation between conservation degree and maximum cysteine thiol reactivity change per protein**. Comparison proteins are binned according to their degree of conservation in response to different stimuli. Correlation and corresponding 95% confidence intervals were derived via automatic bootstrap estimation of the linear regression for either individual stimuli (colored samples) or the comparison dataset as a whole (black dashed line).

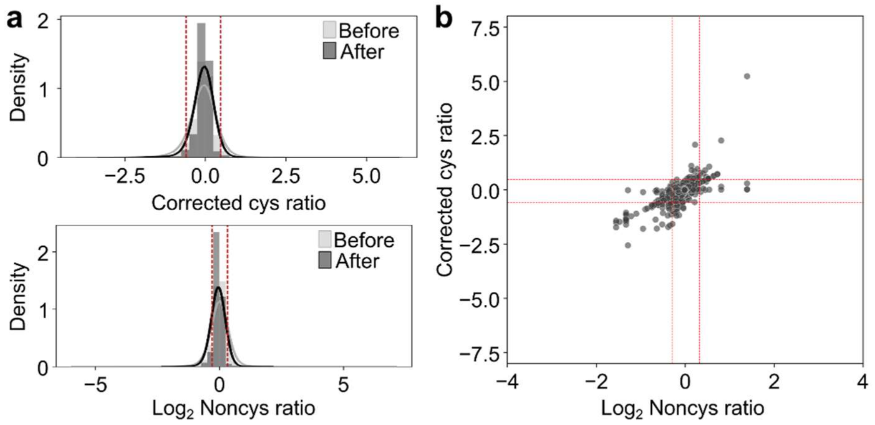

<u>Supplementary Figure 7:</u> Threshold derivation from control dataset. Histograms for the control vs control dataset of (***a***) cysteine and (***b***) noncysteine-containing peptide ratios before and after p-value scaling. The z-score was calculated for each peptide, and thresholds set according to values at which the z-score surpassed 1.96, such that 95% of the control data is contained within the thresholds. (***c***) The resultant thresholded scatterplot for the control dataset.
