# Supplementary material for: Protein painting reveals pervasive remodeling of conserved proteostasis machinery in response to pharmacological stimuli
Source: NPJ Syst Biol Appl. 2022 Nov 28;8:46. doi: 10.1038/s41540-022-00256-3 (PMC9705707; doi:10.1038/s41540-022-00256-3)
Supplement: Supplementary file 2 — Reporting Summary [file 41540_2022_256_MOESM2_ESM.pdf]

nature portfolio

Corresponding author(s): Dezerae Cox
Danny M. Hatters

Last updated by author(s): Oct 7, 2022

Reporting Summary

Nature Portfolio wishes to improve the reproducibility of the work that we publish. This form provides structure for consistency and transparency in reporting. For further information on Nature Portfolio policies, see our Editorial Policies and the Editorial Policy Checklist.

Statistics

For all statistical analyses, confirm that the following items are present in the figure legend, table legend, main text, or Methods section.

n/a	Confirmed	
☐	☒	The exact sample size (*n*) for each experimental group/condition, given as a discrete number and unit of measurement
☐	☒	A statement on whether measurements were taken from distinct samples or whether the same sample was measured repeatedly
☐	☒	The statistical test(s) used AND whether they are one- or two-sided<br>*Only common tests should be described solely by name; describe more complex techniques in the Methods section.*
☒	☐	A description of all covariates tested
☐	☒	A description of any assumptions or corrections, such as tests of normality and adjustment for multiple comparisons
☐	☒	A full description of the statistical parameters including central tendency (e.g. means) or other basic estimates (e.g. regression coefficient) AND variation (e.g. standard deviation) or associated estimates of uncertainty (e.g. confidence intervals)
☐	☒	For null hypothesis testing, the test statistic (e.g. *F*, *t*, *r*) with confidence intervals, effect sizes, degrees of freedom and *P* value noted<br>*Give P values as exact values whenever suitable.*
☒	☐	For Bayesian analysis, information on the choice of priors and Markov chain Monte Carlo settings
☒	☐	For hierarchical and complex designs, identification of the appropriate level for tests and full reporting of outcomes
☒	☐	Estimates of effect sizes (e.g. Cohen's *d*, Pearson's *r*), indicating how they were calculated

Our web collection on statistics for biologists contains articles on many of the points above.

Software and code

Policy information about availability of computer code

Data collection	Raw flow cytometry data were collected using BD FACSDiva Software v 8.0.1<br>Raw mass spectra were obtained using the Xcalibur instrument control software v 4.3.73.11 and Tune application v 3.3.2782.34<br>Protein feature data were programatically compiled on demand using custom analysis scripts available via 10.5281/zenodo.6548916 [https://doi.org/10.5281/zenodo.6548916] from the following database APIs:<br>- UniProt [https://www.uniprot.org/]<br>- Protein Data Bank [https://www.ebi.ac.uk/pdbe/]<br>- STRINGdb [https://www.string-db.org/api/]<br>- PantherGOSlim [http://pantherdb.org]
Data analysis	MaxQuant v 1.6.2.10<br>GraphPad Prism v 8.4.3<br>Python v 3.8.2<br>SciPy v 1.5.0<br>Scikit-learn v 0.23.1<br>Custom analysis scripts available via 10.5281/zenodo.6548916 [https://doi.org/10.5281/zenodo.6548916]

For manuscripts utilizing custom algorithms or software that are central to the research but not yet described in published literature, software must be made available to editors and reviewers. We strongly encourage code deposition in a community repository (e.g. GitHub). See the Nature Portfolio guidelines for submitting code & software for further information.

Data

Policy information about availability of data

All manuscripts must include a data availability statement. This statement should provide the following information, where applicable:

- Accession codes, unique identifiers, or web links for publicly available datasets
- A description of any restrictions on data availability
- For clinical datasets or third party data, please ensure that the statement adheres to our policy

The mass spectrometry proteomics data generated in this study have been deposited in the ProteomeXchange Consortium via the PRIDE partner repository database with the data set identifier PXD033152[https://www.ebi.ac.uk/pride/archive/projects/PXD033152]. Protein structures presented in Fig. 4 are available via the PDB: 6MSB [http://dx.doi.org/10.2210/pdb6msb/pdb]. Preprocessed datasets for the proteomics and flow cytometry are also available from zenodo via 10.5281/zenodo.6439170 [https://doi.org/10.5281/zenodo.6439170].

Human research participants

Policy information about studies involving human research participants and Sex and Gender in Research.

Reporting on sex and gender	*Use the terms sex (biological attribute) and gender (shaped by social and cultural circumstances) carefully in order to avoid confusing both terms. Indicate if findings apply to only one sex or gender; describe whether sex and gender were considered in study design whether sex and/or gender was determined based on self-reporting or assigned and methods used. Provide in the source data disaggregated sex and gender data where this information has been collected, and consent has been obtained for sharing of individual-level data; provide overall numbers in this Reporting Summary. Please state if this information has not been collected. Report sex- and gender-based analyses where performed, justify reasons for lack of sex- and gender-based analysis.*
Population characteristics	*Describe the covariate-relevant population characteristics of the human research participants (e.g. age, genotypic information, past and current diagnosis and treatment categories). If you filled out the behavioural & social sciences study design questions and have nothing to add here, write "See above."*
Recruitment	*Describe how participants were recruited. Outline any potential self-selection bias or other biases that may be present and how these are likely to impact results.*
Ethics oversight	*Identify the organization(s) that approved the study protocol.*

Note that full information on the approval of the study protocol must also be provided in the manuscript.

Field-specific reporting

Please select the one below that is the best fit for your research. If you are not sure, read the appropriate sections before making your selection.

☒ Life sciences ☐ Behavioural & social sciences ☐ Ecological, evolutionary & environmental sciences

For a reference copy of the document with all sections, see nature.com/documents/nr-reporting-summary-flat.pdf

Life sciences study design

All studies must disclose on these points even when the disclosure is negative.

Sample size	Replicates were chosen based on what could be practically handled. We chose no fewer than 3 replicates for each experiment to ensure we could use basic statistical comparisons.
Data exclusions	No data were excluded
Replication	At least three biological replicates were performed for proteomics experiments, and at least four for flow cytometry experiments.
Randomization	Homogenous cell populations were randomly assigned to individual treatment conditions to prevent batch growth effects. Sample labelling strategies for proteomics were not randomised.
Blinding	Proteomics spectra were acquired in a blinded fashion. Initial sample preparations and final analyses were not blinded, however were without any subjective measurement steps.

Reporting for specific materials, systems and methods

We require information from authors about some types of materials, experimental systems and methods used in many studies. Here, indicate whether each material, system or method listed is relevant to your study. If you are not sure if a list item applies to your research, read the appropriate section before selecting a response.

Materials & experimental systems

n/a	Involved in the study
☒	☐ Antibodies
☐	☒ Eukaryotic cell lines
☒	☐ Palaeontology and archaeology
☒	☐ Animals and other organisms
☒	☐ Clinical data
☒	☐ Dual use research of concern

Methods

n/a	Involved in the study
☒	☐ ChIP-seq
☐	☒ Flow cytometry
☒	☐ MRI-based neuroimaging

Eukaryotic cell lines

Policy information about cell lines and Sex and Gender in Research

Cell line source(s)	Neuro2a from American Type Culture Collection
Authentication	Cells were not authenticated
Mycoplasma contamination	Cells were tested negative for mycoplasma
Commonly misidentified lines (See ICLAC register)	None

Flow Cytometry

Plots

Confirm that:

☒ The axis labels state the marker and fluorochrome used (e.g. CD4-FITC).

☒ The axis scales are clearly visible. Include numbers along axes only for bottom left plot of group (a 'group' is an analysis of identical markers).

☒ All plots are contour plots with outliers or pseudocolor plots.

☒ A numerical value for number of cells or percentage (with statistics) is provided.

Methodology

Sample preparation	Following incubation with compounds of interest, media was removed and replaced with a half-volume of fresh serum free media containing TPE-MI to a final concentration of 100 μM. Cells were incubated for 30 min, then immediately washed with 3× excess of PBS containing 10 mM Glutathione to react any remaining TPE-MI. Cells were then washed with PBS, mechanically detached using a cell scraper and centrifuged at 300 g for 5 min. cells were resuspended in PBS and analyzed using an LSRFortessa flow cytometer (BD Biosciences). Between 10,000–60,000 events were collected at high flow rate using a forward scatter threshold of 5000. Pulse area, height and width data were collected with the 355 nm laser and a 450 ± 50 nm bandpass emission filter.
Instrument	BD Biosciences LSRFortessa
Software	BD FACSDiva v 8.0.1
Cell population abundance	Cells were not sorted
Gating strategy	Main cells were gated according to FSC and SSC to include those falling in the central region of the scatterplot. TPE-MI positive cells were then identified by gating to exclude the unstained population according to SSC and TPE-MI fluorescence

☒ Tick this box to confirm that a figure exemplifying the gating strategy is provided in the Supplementary Information.
